# Widespread Dysregulation of Peptide Hormone Release in Mice Lacking Adaptor Protein AP-3

**DOI:** 10.1371/journal.pgen.1003812

**Published:** 2013-09-26

**Authors:** Daniel W. Sirkis, Robert H. Edwards, Cédric S. Asensio

**Affiliations:** 1 Graduate Program in Pharmaceutical Sciences and Pharmacogenomics, University of California San Francisco, San Francisco, California, United States of America; 2 Departments of Physiology and Neurology, University of California San Francisco, San Francisco, California, United States of America; University of Pennsylvania, United States of America

## Abstract

The regulated secretion of peptide hormones, neural peptides and many growth factors depends on their sorting into large dense core vesicles (LDCVs) capable of regulated exocytosis. LDCVs form at the *trans*-Golgi network, but the mechanisms that sort proteins to this regulated secretory pathway and the cytosolic machinery that produces LDCVs remain poorly understood. Recently, we used an RNAi screen to identify a role for heterotetrameric adaptor protein AP-3 in regulated secretion and in particular, LDCV formation. Indeed, *mocha* mice lacking AP-3 have a severe neurological and behavioral phenotype, but this has been attributed to a role for AP-3 in the endolysosomal rather than biosynthetic pathway. We therefore used *mocha* mice to determine whether loss of AP-3 also dysregulates peptide release *in vivo*. We find that adrenal chromaffin cells from *mocha* animals show increased constitutive exocytosis of both soluble cargo and LDCV membrane proteins, reducing the response to stimulation. We also observe increased basal release of both insulin and glucagon from pancreatic islet cells of *mocha* mice, suggesting a global disturbance in the release of peptide hormones. AP-3 exists as both ubiquitous and neuronal isoforms, but the analysis of mice lacking each of these isoforms individually and together shows that loss of both is required to reproduce the effect of the *mocha* mutation on the regulated pathway. In addition, we show that loss of the related adaptor protein AP-1 has a similar effect on regulated secretion but exacerbates the effect of AP-3 RNAi, suggesting distinct roles for the two adaptors in the regulated secretory pathway.

## Author Summary

The physiological action of peptide hormones and neural peptides depends on their sorting to vesicles capable of regulated exocytosis in response to stimulation. Despite the diversity and importance of signals released by this pathway, surprisingly little is understood about the molecular mechanisms involved in sorting to and indeed formation of the large dense core vesicles (LDCVs) that mediate regulated secretion as opposed to secretory vesicles that undergo constitutive release. We recently used RNA interference in cell lines to identify a requirement for the adaptor protein AP-3 in sorting to the regulated secretory pathway, but the importance of this role *in vivo* has remained unknown. Using mutant mice lacking various subunits of the AP-3 complex, we now show that AP-3 is indeed required for appropriate, regulated secretion in multiple neuroendocrine cell types. Although AP-3 exists as both ubiquitous and neuronal forms, we also find that either form alone suffices to confer regulated secretion. The results show that AP-3 plays a novel and essential role in regulating the release of peptide hormones and neural peptides.

## Introduction

In contrast to most proteins which undergo immediate and unregulated secretion after biosynthesis, proteins destined for regulated release require sorting into LDCVs, but the mechanisms responsible for sorting to LDCVs and indeed LDCV formation remain poorly understood. LDCVs bud from the *trans*-Golgi network (TGN) [1], [2], [3], and previous work has suggested that lumenal interactions such as the aggregation of granulogenic proteins drive their formation [4], [5]. Indeed, sorting to LDCVs has been suggested to occur by default, with proteins destined for other organelles removed during the well-established process of LDCV maturation [6], [7]. However, direct analysis of budding from the TGN has demonstrated the sorting of regulated from constitutive cargo at this early step, before maturation [3]. In addition, LDCV membrane proteins such as carboxypeptidase E and sortilin have been proposed to serve as the receptors for soluble cargo [8], [9]. In contrast to these lumenal and membrane interactions, the cytosolic machinery involved in sorting to LDCVs and LDCV formation has remained poorly understood.

Several membrane proteins contain cytosolic sequences that direct them to LDCVs. For example, the neuronal vesicular monoamine transporter VMAT2, which fills neurosecretory vesicles with monoamine transmitter, depends on a conserved, C-terminal, cytoplasmic dileucine-like motif for sorting to LDCVs [10], [11], [12], and the LDCV membrane protein IA-2β (phogrin) relies on a remarkably similar sequence [13]. Since the requirement for a cytoplasmic motif suggested an interaction with cytosolic sorting machinery, we recently used VMAT as a reporter to screen by RNAi in *Drosophila* S2 cells for proteins involved in biogenesis of the regulated secretory pathway, identifying multiple subunits of the heterotetrameric adaptor protein AP-3 [14]. Loss of AP-3 results in mis-sorting of VMAT in both S2 and mammalian neuroendocrine PC12 cells, dysregulated secretion, a reduction in the number and alteration in the morphology of LDCVs [14]. Indeed, AP-3 RNAi disrupts sorting at the TGN and impairs the concentration of membrane proteins such as synaptotagmin that are required for regulated release [14]. However, most work in mammalian cells has focused on the role of AP-3 within the endolysosomal pathway, in trafficking from early endosome to lysosome.

Consistent with a role in the endolysosomal pathway, *mocha* mice (*Mus musculus*) lacking AP-3 show defects in lysosome-related organelles (LROs) such as melanosomes, platelet granules and synaptic vesicles [15], [16]. In addition to the abnormal coat color and a bleeding diathesis, however, the animals exhibit perinatal lethality, hyperactivity, head tilt, seizures and reduced fertility [17], [18], [19], and it has remained unclear whether a defect in the endolysosomal pathway can fully account for the severe phenotype. Importantly, previous work in *S. cerevisiae* has indicated a primary role for AP-3 in the biosynthetic pathway [20], [21], [22]. We have thus now used *mocha* mice to investigate the physiological role of mammalian AP-3 in regulated protein secretion.

## Results

### The *mocha* Mutation Dysregulates Release by Adrenal Chromaffin Cells

To determine whether the loss of AP-3 *in vivo* affects regulated secretion, we cultured adrenal chromaffin cells from control and AP-3-deficient *mocha* mice, measuring the release of endogenous secretogranin II (SgII) in response to the nicotinic agonist DMPP [23]. Western blotting of the medium indicated that DMPP stimulates SgII secretion from control cells, but SgII was undetectable in the medium of *mocha* cells (Figure S1). However, the substantial reduction in cellular SgII content of *mocha* adrenal glands [14] and of cultured *mocha* adrenal chromaffin cells (Figure S1) made it difficult to determine whether the cells simply do not contain and release enough SgII to detect, or actually exhibit a defect in regulated release.

**Figure 1:**
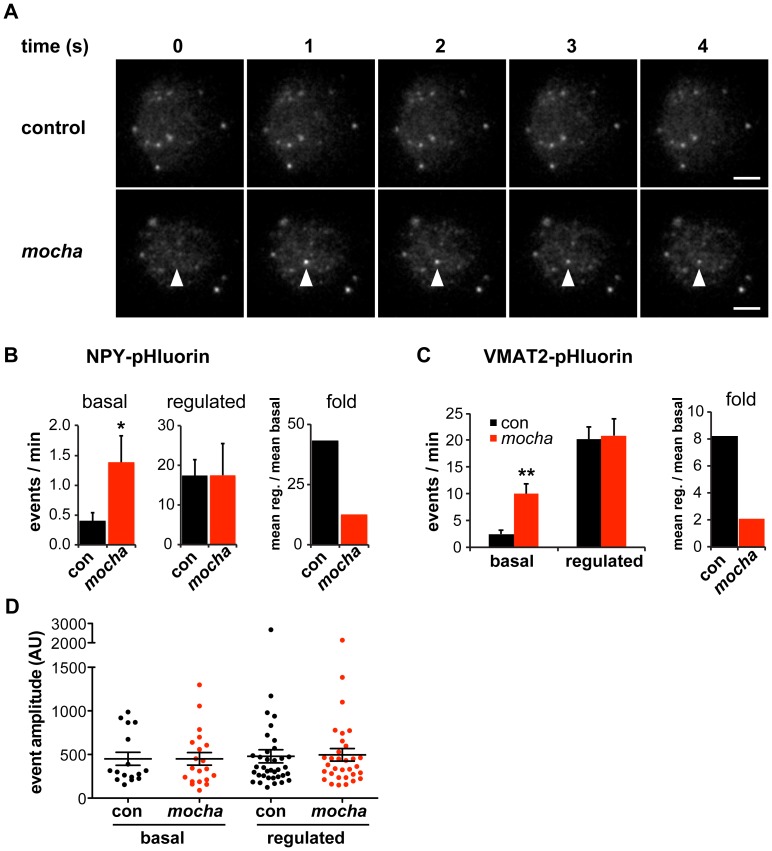
*mocha* chromaffin cells display dysregulated release of NPY and VMAT2. Chromaffin cells were transduced with lentivirus encoding either NPY- or VMAT2-pHluorin, then imaged live 4–7 days later by TIRF microscopy. Basal exocytosis was measured in Tyrode's solution, and release stimulated in Tyrode's containing 5 µM DMPP. (A) Representative images acquired during the unstimulated phase show a typical basal exocytotic event in a *mocha* cell. Scale bars indicate 5 µm. (B) Quantification of individual exocytotic events shows increased basal NPY-pHluorin exocytosis in *mocha* cells, and a similar number of events relative to control cells after stimulation. *p<0.04 relative to control; for basal, n = 38 for control and n = 38 for *mocha*; for stimulated, n = 22 for control and n = 22 for *mocha*. Normalizing mean regulated events to mean basal events shows that *mocha* cells have an ∼70% reduction in secretion fold change in response to stimulation relative to control cells. (C) Quantification of exocytotic events as above shows increased basal VMAT2-pHluorin exocytosis in *mocha* cells. **p<0.0003 relative to control; n = 18 for control and n = 16 for *mocha*. Analysis of the secretion fold change shows that *mocha* cells have an ∼75% reduction relative to control. (D) Analysis of the amplitude of exocytotic events monitored using NPY-pHluorin reveals that basal and regulated events of both control and *mocha* cells are comparable in size. The bars indicate mean values, and error bars the s.e.m.

To assess regulated exocytosis by chromaffin granules, we used total internal reflection fluorescence (TIRF) microscopy to image neuropeptide and LDCV membrane protein reporters fused to the superecliptic pHluorin [24], [25]. The pHluorin is a modified form of green fluorescent protein (GFP) with increased sensitivity to protons that is quenched at the low internal pH of LDCVs and therefore increases in fluorescence with exposure to the higher external pH on exocytosis. Since neuropeptide Y (NPY)-pHluorin has been shown to undergo regulated exocytosis [26], we used lentiviral transduction to express this fusion protein and monitored individual exocytotic events at the plasma membrane of living chromaffin cells. In the absence of stimulation, control cells showed very few spontaneous fusion events over 90 s of imaging, but AP-3-deficient *mocha* cells exhibited substantially more (Figure 1A–1B). Both control and *mocha* cells showed a clear increase in exocytosis in response to stimulation by DMPP, but the extent of stimulation relative to baseline reveals an ∼70% reduction in *mocha* cells compared to controls (Figure 1B). To extend these findings to an LDCV membrane protein, we transduced chromaffin cells with a virus encoding VMAT2-pHluorin, with the lumenal location of pHluorin enabling detection of release events [27], [28]. Similar to NPY-pHluorin, VMAT2-pHluorin also showed a clear increase in basal, unstimulated exocytosis in *mocha* relative to control cells, and this again resulted in an ∼75% reduction in stimulated release (Figure 1C). The loss of AP-3 thus dysregulates the release of LDCVs as monitored using either soluble or membrane cargo.

The role of AP-3 in defining LDCV membrane protein composition has suggested that loss of the adaptor results in mixing of constitutive and regulated secretory pathways [14]. The resulting constitutive secretion (measured biochemically in the case of endogenous SgII [14]) could thus reflect either the spontaneous release of constitutive secretory vesicles which contain mis-sorted LDCV cargo but no dense core, or the dysregulated release of LDCVs. To distinguish between these possibilities, we again took advantage of TIRF microscopy. Release from constitutive vesicles without a dense core should yield events with reduced amplitude relative to controls, whereas dysregulated LDCV fusion should yield events with a size similar to controls. Analyzing individual exocytotic events, we observed that the basal as well as stimulated events observed in *mocha* cells have an amplitude similar to those observed in controls (Figure 1D). The heightened basal secretion observed in *mocha* cells thus apparently results from dysregulated exocytosis of LDCVs rather than the fusion of constitutive secretory vesicles without a dense core.

### Dysregulated Peptide Hormone Release by Pancreatic Islet Cells from *mocha* Mice

**Figure 2:**
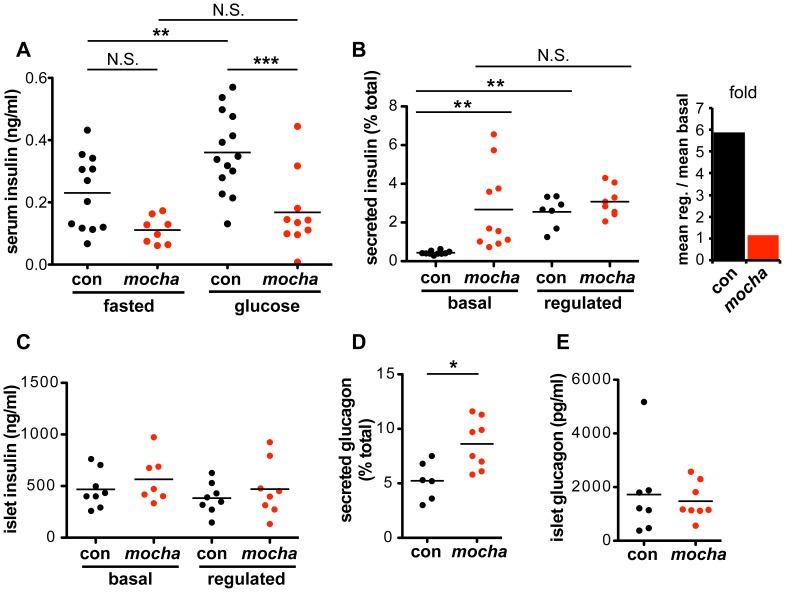
*mocha* mice show dysregulated secretion of insulin and glucagon. (A) Serum insulin levels were determined after an overnight fast (baseline) and 15–20 min after i.p. injection of glucose (2 mg/g body weight). In contrast to the controls, *mocha* insulin levels do not significantly increase after glucose injection. The data derive from two independent experiments; n = 14 control and n = 10 *mocha* mice. **p<0.01; ***p<0.001 by Newman-Keuls post-hoc test. Pancreatic islets were isolated acutely and bathed in either 2.8 mM (basal) or 28 mM (regulated) glucose for 1 h at 37°C. (B) *mocha* islets display markedly increased basal insulin secretion compared to controls. In contrast, control and *mocha* islets secrete comparable amounts of insulin in response to high glucose. For control islets, this represents an ∼6-fold increase in secretion in response to glucose elevation. For *mocha* islets, insulin secretion is not significantly increased in response to glucose elevation. **p<0.01 by Dunnett's post-hoc test, n = 7–10. (C) After collecting secreted insulin, the islets were pelleted and lysed by sonication to extract cellular insulin. Cellular insulin levels are similar between all groups. (D) Basal glucagon secretion measured in 28 mM glucose is significantly increased by the *mocha* mutation. (E) Cellular glucagon levels do not differ between *mocha* and control mice. *p<0.02, n = 6–8. The bars represent mean values.

The increased basal exocytosis of NPY and VMAT2 from *mocha* chromaffin cells is consistent with earlier experiments using RNAi in PC12 cells [14], but does the loss of AP-3 also dysregulate release from other neuroendocrine tissues? Pancreatic β cells store insulin in LDCVs morphologically and biochemically similar to chromaffin granules [29], [30]. To assess the regulated release of insulin *in vivo*, we measured baseline serum insulin levels while fasting and stimulated levels 15–20 minutes after intraperitoneal injection of glucose (Figure 2A). Before glucose administration, we observed a slight reduction in the serum insulin levels of *mocha* mice relative to controls, but this did not reach significance. After glucose administration, the control animals showed a clear increase in serum insulin but the *mocha* mice did not (Figure 2A), suggesting a failure of regulated release.

To assess the consequences of dysregulated insulin release for carbohydrate metabolism, we also measured blood glucose. Surprisingly, we observed no clear difference in fasting blood glucose levels between control and *mocha* mice (Figure S2A). After glucose administration, *mocha* animals show an increase in glycemia but less than controls (Figure S2A), a surprising effect in light of the lower serum insulin levels which would have been expected to impair glucose tolerance (Figure 2A). The effects of the *mocha* mutation on blood glucose levels thus do not correlate with the effects on insulin. However, blood glucose levels reflect the combined action of multiple circulating hormones, many of which may be affected by the loss of AP-3. Indeed, the dysregulated release of other peptide hormones may indirectly affect the release of insulin.

To examine insulin release independent of systemic effects, we isolated pancreatic islets and acutely incubated them in medium containing either low or high concentrations of glucose. Figure 2B shows that in contrast to the clear stimulation of insulin release by high concentrations of glucose in control islet cells, *mocha* cells exhibit increased basal release with little if any stimulation by high glucose. As opposed to the reduced content of SgII in *mocha* chromaffin cells, cellular insulin levels show no difference between *mocha* and control islets (Figure 2C), indicating that the change in basal secretion is not secondary to altered expression of the hormone. In the case of insulin, the *mocha* mutation thus dramatically and selectively impairs regulated secretion.

Since the effects of the *mocha* mutation on release of other peptide hormones may complicate the observations *in vivo*, we also examined glucagon, a peptide released from pancreatic α cells that opposes the action of insulin: glucagon raises blood glucose levels in response to starvation. Although we observed no effect of the *mocha* mutation on fasting serum glucagon (Figure S2B), baseline glucagon secretion was increased in acutely isolated islets (Figure 2D). In addition, the glucagon content of *mocha* islet cells did not differ from controls (Figure 2E), and the morphology of the islets *in situ* appears unchanged (Figure S2C). Thus, *mocha* mice show dysregulated release of two peptide hormones with opposing actions, suggesting a global effect on peptide hormone release that makes it difficult to predict the net consequences for glucose homeostasis *in vivo*.

### Loss of Both Ubiquitous and Neural AP-3 Isoforms Is Required to Recapitulate the LDCV Phenotype of *mocha* Mice

**Figure 3:**
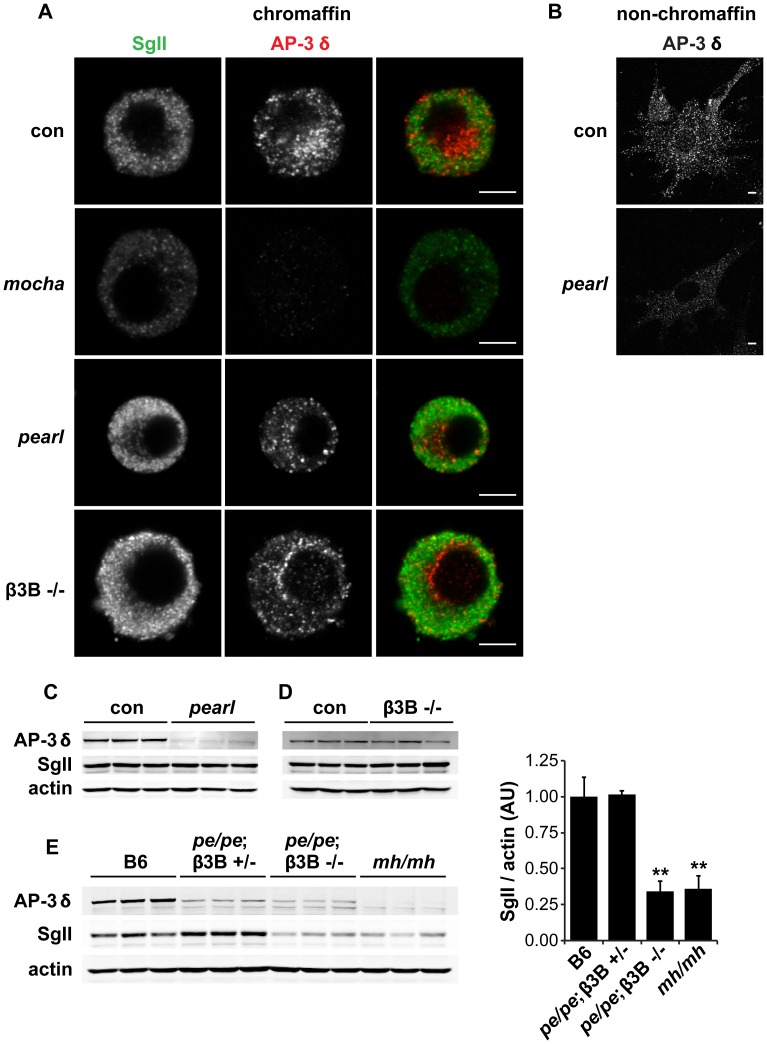
Concomitant loss of both β3A and β3B is required to reduce adrenal SgII. (A) Effect of individual AP-3 subunit mutations on SgII and AP-3 δ levels by immunofluorescence. Adrenal chromaffin cells from *mocha* (δ), *pearl* (β3A), β3B−/−, and control mice were stained with a rabbit polyclonal antibody to SgII and a mouse monoclonal antibody to AP-3 δ, followed by an anti-rabbit antibody conjugated to Alexa Fluor 488 and an anti-mouse antibody conjugated to Alexa Fluor 594. Representative confocal micrographs show the expected reduction in SgII and absence of AP-3 δ staining in *mocha* chromaffin cells, but unchanged SgII and AP-3 δ in both *pearl* and β3B−/− cells. (B) Non-chromaffin, fibroblast-like *pearl* cells present in the culture show a clear reduction in AP-3 δ. Scale bars indicate 5 µm. (C) The adrenal glands of *pearl* mice show a clear reduction in overall AP-3 levels, but unchanged SgII. (D) Adrenal glands of β3B−/− mice show no change in overall AP-3 or SgII levels. (E) Adrenal glands of double mutant *pe/pe*; β3B−/− mice show a clear reduction in SgII levels relative to age-matched C57BL/6 controls and *pe/pe*; β3B+/− controls. *mocha* adrenals show a reduction in SgII similar to that in *pe/pe*; β3B−/− mice. **p<0.001 relative to control by Dunnett's post-hoc test, n = 3. The data show mean values, and error bars indicate s.e.m.

The heterotetrameric AP-3 complex is known to exist in two isoforms, one expressed by all tissues, and another expressed more specifically by neurons and endocrine tissue including the adrenal gland and pancreatic islets [16], [31], [32]. In metazoan cells, the ubiquitous isoform contributes to trafficking from early endosomes to the lysosome through a pathway that does not involve multivesicular bodies [15], [33]. In contrast, the neural isoform has been implicated in the formation of synaptic vesicles from an endosomal intermediate [34], [35], suggesting that this isoform may also contribute to the formation of LDCVs. To test this possibility, we used *pearl* mice lacking the ubiquitous isoform of the β3 subunit (β3A) and β3B knockouts lacking the neural isoform of β3. Since the δ subunit of AP-3 exists as only a single isoform, and the loss of one subunit usually destabilizes the complex [17], [36], we first stained cells in culture for δ to assess the effect of the mutations on the complex as a whole. We were surprised to observe no effect of losing either β3 isoform on the levels of immunoreactive δ in chromaffin cells (Figure 3A), particularly considering the abundance of the ubiquitous β3A subunit in most tissues. However, we did observe reduced expression of δ by β3A-deficient, non-chromaffin cells in the culture (Figure 3B), presumably because they do not express the neural isoform and therefore cannot exhibit redundancy. Consistent with the relative abundance of adrenal cortical cells [37] and of the ubiquitous AP-3 isoform, western analysis of adrenal homogenates showed low levels of AP-3 δ in β3A-deficient *pearl* animals (Figure 3C) but normal levels in β3B knockouts (Figure 3D).

To determine whether the loss of β3A or β3B influences the formation of LDCVs, we then examined the effects on SgII. We were surprised to observe that in contrast to *mocha* cells which showed the reduction previously reported [14], both β3 mutants had normal levels of SgII by immunofluorescence (Figure 3A). By western analysis of adrenal extracts, both β3A-deficient *pearl* and β3B knockouts also contained normal levels of immunoreactive SgII (Figure 3C–3D). However, loss of both isoforms in the adrenal gland of double mutant mice produced a reduction in SgII comparable to that observed in *mocha* mice (Figure 3E). With regard to the cellular content of SgII, the two isoforms thus exhibit redundancy.

### Reduced Granin Content Reflects Reduced Gene Expression but Not Increased Degradation

**Figure 4:**
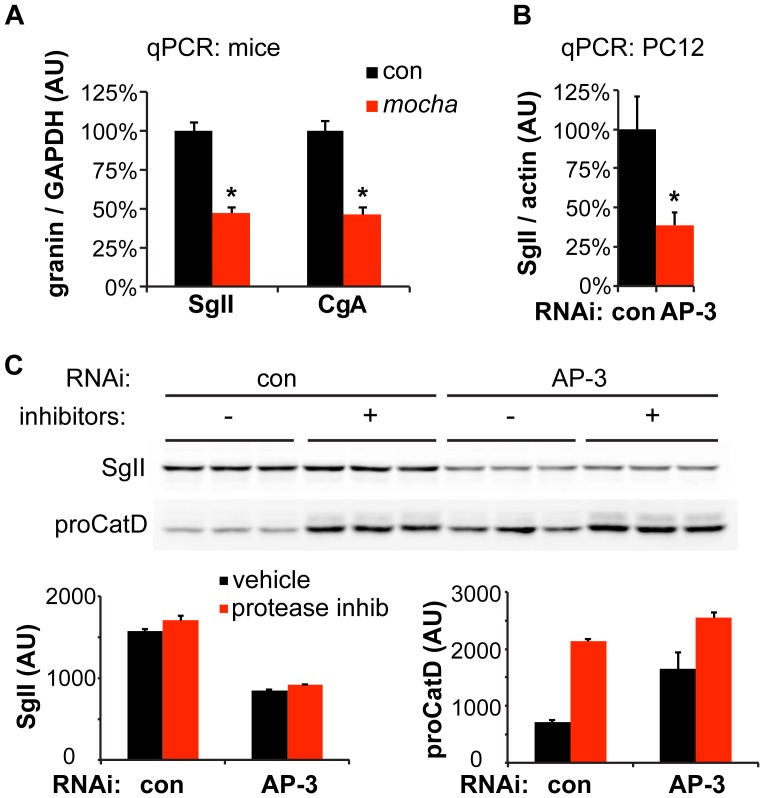
Loss of AP-3 reduces granin mRNA but does not increase its degradation. (A) Quantitative RT-PCR (qPCR) analysis of adrenal gland transcripts from *mocha* mice shows an ∼50% reduction in SgII and CgA expression relative to the adrenals of littermate controls. *p<1×10^−4^, n = 6–7 adrenals per genotype. (B) qPCR analysis of PC12 cells transfected with AP-3 δ siRNA shows an ∼60% reduction in SgII expression relative to cells transfected with control siRNA. *p<0.02, n = 5–6 transfections. (C) Incubation of PC12 cells with lysosomal protease inhibitors for ∼24 h has no effect on cellular SgII levels in cells transfected with either AP-3 δ or control siRNA. In contrast, the protease inhibitors markedly elevate the levels of the lysosomal hydrolase precursor procathepsin D. The data show mean values, and error bars indicate s.e.m.

The reduced expression of SgII in mice lacking AP-3 might reflect increased basal secretion or an entirely distinct process. LDCV contents have indeed been shown to undergo transcriptional regulation through a variety of mechanisms [30], [38]. We therefore measured adrenal SgII and chromogranin A (CgA) transcripts from control and *mocha* adrenals by quantitative reverse transcription (qRT)-PCR. Both SgII and CgA mRNA were substantially reduced (by ∼50%) in *mocha* mice, although not to the same extent as the protein [14] (Figure 4A). PC12 cells showed a similar reduction in SgII mRNA after AP-3 RNAi (Figure 4B).

Since AP-3 influences trafficking within the endolysosomal pathway, loss of the adaptor may also influence SgII levels through increased degradation in the lysosome. To test this possibility, we inhibited lysosomal proteases after AP-3 knockdown in PC12 cells, but did not observe any increase in the levels of SgII (Figure 4C). On the other hand, the level of lysosomal hydrolase precursor procathepsin D dramatically increased in response to the inhibition of lysosomal degradation, indicating the effectiveness of the inhibitors (Figure 4C). The reduction in cellular SgII observed with loss of AP-3 thus reflects reduced expression as well as increased constitutive release, but not increased degradation.

### Non-redundant Roles for AP-3 and AP-1 in the Regulated Secretory Pathway

**Figure 5:**
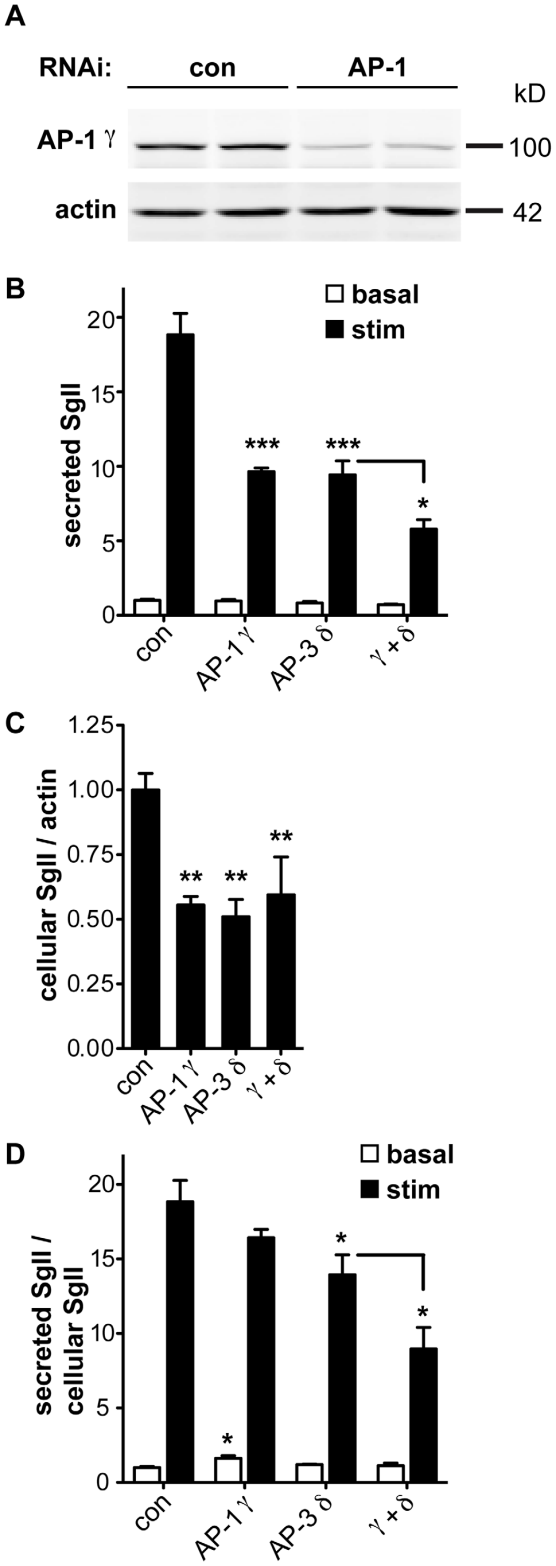
AP-1 knockdown potentiates the effect of AP-3 knockdown on regulated secretion. (A) PC12 cells were transfected with pooled siRNA directed against AP-1 γ or pooled nontargeting siRNA as control. Knockdown was assessed by quantitative fluorescent immunoblotting for AP-1 γ with actin as loading control. (B) PC12 cells were transfected with control, AP-1 γ, AP-3 δ or both siRNAs, washed, and incubated for 30 min in Tyrode's solution containing 2.5 mM (basal) or 50 mM (stimulated) K^+^. Secreted SgII was measured by quantitative fluorescent immunoblotting and normalized to basal secretion from cells treated with control siRNA. AP-1 γ RNAi alone reduces the depolarization-induced secretion of SgII very similar to AP-3 δ RNAi, and combined AP-1 γ/AP-3 δ RNAi potentiates the decrease in regulated secretion. (C) Cells were lysed and intracellular SgII measured as above, normalizing to actin. AP-1 γ and AP-3 δ RNAi both reduce cellular SgII, but the combined knockdown does not show an additional effect. (D) Secreted SgII was normalized to cellular SgII levels for each RNAi condition, and expressed relative to basal secretion from cells treated with control siRNA. Normalizing to cellular SgII shows a significant increase in basal secretion of SgII with AP-1 γ RNAi whereas AP-3 δ RNAi significantly reduces depolarization-induced secretion. Combined AP-1 γ/AP-3 δ RNAi potentiates the reduction in stimulated release. *p<0.05; **p<0.01; ***p<0.001 (Newman-Keuls post-hoc test; n = 4 transfections). The data show mean values, and error bars indicate s.e.m.

AP-3 resembles AP-1 in terms of sequence, the ability to recognize similar trafficking motifs and subcellular location at endosomes and the Golgi apparatus [39], [40], [41]. In addition, AP-1 associates with immature LDCVs and promotes their maturation through the clathrin-dependent removal of proteins destined for other organelles [6], [42]. In mouse pituitary AtT-20 cells, maturation indeed contributes to regulated release by removing the inhibitory protein synaptotagmin 4 [43]. In PC12 and pancreatic islet cells, however, immature LDCVs can undergo regulated release [44], [45]. To determine whether silencing of AP-1 impairs regulated release from PC12 cells, we initially targeted AP-1 β-adaptin since this is the only mammalian AP-1 subunit without multiple isoforms and hence with reduced potential for redundancy. Despite highly efficient knockdown of the β1 subunit by RNAi, μ1A levels were not reduced (data not shown), raising the possibility that the β2 subunit (of AP-2) stabilized the complex by replacing β1 [46], [47]. We therefore targeted the γ subunit of AP-1, in particular the γ1 isoform which appears to show little redundancy with γ2 [48]. siRNA transfection reduced endogenous γ1 protein by ∼80% (Figure 5A). It also reduced the stimulated secretion of SgII and the intracellular accumulation of SgII, very similar to AP-3 δ RNAi (Figure 5B,C). However, normalizing to the reduced cellular stores of SgII revealed more of an increase in basal SgII secretion than a reduction in stimulated release with AP-1 knockdown, in contrast to AP-3 RNAi (Figure 5D). AP-1 RNAi thus still reduces the extent of stimulation (stimulated/basal release) from 19-fold for control to 10-fold for AP-1 RNAi (p<0.005). AP-1 knockdown also potentiates the effect of AP-3 RNAi on stimulated secretion even after normalization to the reduced intracellular stores of SgII (Figure 5D). AP-1 thus contributes to regulated secretion, and its role appears independent at least in part from that of AP-3.

## Discussion

The results show that *mocha* mice have a major defect in the regulated secretion of peptide hormones. *mocha* animals exhibit hyperactivity, poor fertility, seizures and premature lethality [18], [19], but this has generally been attributed to a role for AP-3 in the endolysosomal pathway and the formation of LROs [15], [34]. We now find that *mocha* animals exhibit dysregulated exocytosis of adrenal chromaffin granules and both insulin- and glucagon-containing granules from pancreatic islet cells. All of the *mocha* cells examined show increased constitutive release relative to their cellular stores. In addition, they all show a reduced effect of stimulation, with a virtually complete loss of regulation in pancreatic β cells. Consistent with these findings in *mocha* mice, AP-3 RNAi increases constitutive and reduces stimulated LDCV release in PC12 cells [14]. Considering the parallel effects of AP-3 deficiency on LDCV behavior in chromaffin and pancreatic islet as well as PC12 cells, we infer that the dysregulated release observed in *mocha* mice reflects a common disturbance in the formation of LDCVs. The dysregulation of release by multiple neuroendocrine populations further suggests that a global defect in regulated protein secretion contributes to the phenotype of *mocha* mice, although the specific peptides contributing to individual features of the phenotype such as hyperactivity and seizures remain unknown.

In previous work, we found that the loss of AP-3 reduces the amount of SgII stored in PC12 cells and the adrenal gland [14]. This reflects the increased baseline exocytosis of LDCVs, but we now find that multiple granin mRNAs down-regulate as well, indicating unanticipated transcriptional effects of AP-3 deficiency. Indeed, the transcriptional down-regulation of certain LDCV cargo may account for the apparently different effects of AP-3 deficiency on different reporters and in different cells. The down-regulation of SgII mRNA in PC12 cells presumably makes it difficult to detect an increase in the absolute amount of SgII released constitutively, but transfection into PC12 cells of pHluorin-based reporters, which are not subject to this down-regulation, reveal the increased basal secretion [14]. We observe a similar increase in basal exocytosis of VMAT2- and NPY-pHluorin expressed in AP-3-deficient chromaffin cells using a lentivirus. In the case of pancreatic islets from *mocha* mice, cellular levels of insulin do not fall, presumably enabling us to detect the increase in basal insulin release.

Similar to AP-3 deficiency, loss of the major LDCV protein IA-2 reduces expression of multiple LDCV cargo [49], [50], suggesting that AP-3 may sort IA-2 to LDCVs. In the absence of AP-3, decreased LDCV IA-2 may thus result in reduced granin gene expression. Although AP-3 has a role in the endolysosomal pathway, we also find that the reduced granin content does not reflect increased degradation. The role for AP-3 in regulated secretion thus appears distinct from its well-established role in trafficking to the lysosome.

The analysis of isoform-specific knockouts indicates redundancy between the ubiquitous and neural isoforms of AP-3 with regard to LDCV formation. Using SgII as a reporter for a defect in the regulated secretory pathway, we find that only the loss of both ubiquitous β3A and neural β3B causes a reduction in adrenal SgII levels. However, the loss of these isoform-specific subunits has differential effects on other trafficking phenomena. In neurons, loss of β3B mimics the effect of the full *mocha* mutation, with reduced presynaptic expression of proteins such as the zinc transporter ZnT3 and the chloride carrier ClC-3 [35]. Loss of β3A, on the other hand, increases presynaptic expression of these proteins [35]. The redundancy of neural and ubiquitous AP-3 forms in LDCV formation thus contrasts with the opposing roles of the two isoforms in delivery of specific proteins to the nerve terminal.

How does AP-3 promote regulated secretion? In PC12 cells, the loss of AP-3 reduces the number of LDCVs and changes their morphology [14]. They appear less dense by gradient fractionation and larger by electron microscopy (EM). Consistent with this, previous work in *mocha* mice has shown enlarged chromaffin granules by amperometry and EM [31]. In addition, we found that AP-3 deficiency affects the membrane proteins required for regulated exocytosis: the calcium sensor synaptotagmin 1 shifts from LDCVs to lighter membranes [14]. An assay for budding from the TGN further shows that AP-3 deficiency impairs LDCV formation [14]. Despite the importance of AP-3 for LDCV formation, however, its role may be indirect, and previous work has indeed localized AP-3 primarily to endosomes [33], [41].

Several observations have suggested a role for AP-3 at the Golgi complex. In yeast, AP-3 contributes to a direct pathway from the Golgi to the vacuole [20], [22], [51]. Although this has been considered specific to yeast, work in mammalian cells has more recently supported a role for AP-3 in delivery of membrane proteins from the biosynthetic pathway to the lysosome [52], [53]. Biochemical studies have further demonstrated the specific binding of AP-3 to membranes derived from the Golgi or to artificial membranes containing the Golgi lipid phosphatidylinositol-4-phosphate (PI4P) [54], [55]. Ultrastructural studies with immunogold have also demonstrated a small pool of AP-3 at the Golgi complex [33], [41]. However, it remains possible that the role of AP-3 in LDCV formation is indirect, helping to recycle critical LDCV membrane proteins to the Golgi, or adding these proteins to LDCVs during their maturation [56].

Despite the complete loss of AP-3 and increased basal secretion, adrenal chromaffin cells from *mocha* mice still show residual stimulated release, raising the possibility that another system also contributes to LDCV formation. Interestingly, previous work has implicated the related adaptor AP-1 in the formation of secretory granules by other cell types, such as glue granules of the *Drosophila* exocrine salivary gland, and the Weibel-Palade bodies of mammalian endothelial cells [57], [58]. AP-1 also has a clear role in LDCV maturation [6], [42], but immature LDCVs can undergo release from PC12 cells [45]. We were therefore surprised to find that loss of AP-1 impairs regulated release by PC12 cells. It remains possible that maturation promotes regulated secretion even if it is not absolutely required. Alternatively, AP-1 may promote regulated release independent of LDCV maturation.

We also find that the loss of AP-1 exacerbates the dysregulation of release by AP-3 RNAi, suggesting that the two adaptors act through distinct mechanisms. If AP-1 promotes regulated secretion through its role in LDCV maturation, it may indeed act to remove proteins that interfere with regulated release, a process that occurs in AtT-20 cells [43]. We speculate that a proofreading role for AP-1 may become even more important in the absence of AP-3 to concentrate the membrane proteins required for regulated secretion.

## Materials and Methods

### Ethics Statement

All procedures involving animals were approved by the UCSF Institutional Animal Care and Use Committee.

### Antibodies

The rabbit SgII antibody was obtained from Meridian Life Science, the mouse actin monoclonal antibody from Millipore, the mouse δ SA4 monoclonal antibody from the Developmental Studies Hybridoma Bank, the goat cathepsin D antibody from Santa Cruz, the mouse HA.11 monoclonal antibody from Covance, the mouse adaptin γ monoclonal antibody from BD Transduction Laboratories, the mouse insulin monoclonal antibody from Sigma, the guinea pig glucagon antibody from Linco, the mouse glucagon monoclonal antibody from Sigma and the rabbit somatostatin antibody from Thermo Scientific.

### siRNAs

*Silencer Select* rat Ap3d1 (sense, 5′-CAUGGAUCAUGACCAAGAA-3′) and corresponding non-targeting control siRNAs were from Ambion. ON-TARGET*plus* rat Ap1g1 (sense, 5′-CAUAAAUAUUCUUGGUCGA-3′, 5′-GUGUGGAGAUGCACGCUUA-3′, 5′-UGUAACAGUGAUAACGAUA-3′, 5′-GGACUGGAAUUCACGGCAA-3′) and corresponding non-targeting pooled control siRNAs were from Dharmacon.

### Molecular Biology

The sequences of NPY-pHluorin (a generous gift of R. Holz, U. Michigan) and VMAT2- pHluorin were amplified by PCR to add 5′ BamHI and 3′ EcoRI sites, then subcloned into the FUGW lentiviral expression vector, replacing the EGFP coding sequence.

### Cell Culture and Lentivirus Production

PC12 cells were maintained in DMEH-21 medium supplemented with 10% horse serum (HS) and 5% cosmic calf serum (CCS; HyClone) in 5% CO_2_ at 37°C. siRNA transfection was performed using Lipofectamine 2000 (Invitrogen) according to the manufacturer's instructions. HEK293T cells were maintained in DMEH-21 medium with 10% fetal bovine serum (FBS) in 5% CO_2_ at 37°C. Lentivirus was produced by transfecting HEK293T cells with FUGW, psPAX2 and pVSVG and Fugene HD (Roche) according to the manufacturer's instructions [59].

### Chromaffin Cell Isolation and Culture

Mouse adrenal chromaffin cells were isolated and cultured as previously described [60]. Briefly, adrenal glands were dissected and placed in cold Ca^++^-, Mg^++^-free (CMF) Hank's balanced salt solution (HBSS). The surrounding fat and cortex were removed and the medullae transferred to tubes containing 300 U/ml Collagenase I (Worthington) in CMF-HBSS. Medullae were dissociated by shaking for 40 min at 37°C. Collagenase solution was then replaced by CMF-HBSS containing 200 µg/ml DNAse I (Sigma) and 1% heat-inactivated FBS (Gibco), the tissue triturated first with a P200 pipette tip, then with a 23 gauge needle. The cells were pelleted at 300 *g* for 8 min at room temperature and resuspended in pre-warmed culture medium. Cells were maintained in DMEH-21 medium supplemented with 10% FBS and antibiotics. For lentiviral transduction, freshly isolated chromaffin cells were plated in viral supernatant, and fresh medium was added the following morning.

### Total Internal Reflection Fluorescence (TIRF) Microscopy

For TIRF microscopy, control or *mocha* chromaffin cells were plated onto glass chambers coated with poly-L-lysine, immediately transduced with lentivirus encoding either NPY- or VMAT2-pHluorin and imaged live 4–7 days later. Images were typically collected for 40–50 ms at 10 Hz and room temperature using an inverted TIRF microscope (TE2000E; Nikon) with 100× Plan Apo 1.49 NA oil objective, a 1.5× tube lens and an electron-multiplying charge-coupled device camera (QuantEM; Photometrics). Basal exocytosis was measured in Tyrode's solution containing (in mM, 119 NaCl, 25 HEPES-NaOH, pH 7.4, 30 glucose, 2.5 KCl, 2 CaCl_2_, 2 MgCl_2_) over 90 s, and release stimulated for 60 s in Tyrode's containing 5 µM 1,1-Dimethyl-4-phenylpiperazinium (DMPP; Sigma). Individual exocytotic events were quantified manually using NIS-Elements software (Nikon). The amplitude of individual exocytotic events was measured by placing 2×2 pixel ROIs manually over the center of events, and the mean ROI intensity prior to an event subtracted from the maximum event intensity.

### Glucose Tolerance Tests and Serum Insulin Measurements

Glucose tolerance was assessed and serum insulin levels measured using 5–15 week-old *mocha* mice and age-matched controls. Mice were fasted overnight (∼16 h), weighed the following morning, and blood samples collected for baseline glucose and insulin levels. Mice were then injected intraperitoneally with glucose at 2 mg/g body weight and blood samples collected from the tail vein at the time points indicated. Blood glucose levels were measured using the FreeStyle glucometer (Abbott). To measure serum insulin, the blood was allowed to clot, sedimented at 2000 *g* and 4°C for 20 min, and insulin levels determined using the Ultra Sensitive Mouse Insulin ELISA kit (Crystal Chem).

### Pancreatic Islet Isolation and Insulin Secretion

Islets were isolated as previously described from 9–16 week-old *mocha* mice and age-matched controls [61]. Briefly, islets were purified on a Ficoll gradient and allowed to recover for 1 h at 37°C. Five islets were aliquoted into tubes containing HEPES-buffered RPMI medium supplemented with either 2.8 mM glucose (basal) or 28 mM glucose (regulated) and incubated for 1 h at 37°C. The islets were then sedimented and the supernatants collected to measure insulin secretion. The pellets were resuspended and sonicated in 2 mM acetic acid, 0.25% RIA-grade BSA to extract intracellular insulin. Finally, the nuclei were lysed by additional sonication in 67 mM ammonium hydroxide, 0.2% Triton X-100. Secreted and cellular insulin were quantified by ELISA (Mercodia) according to the manufacturer's instructions, and islet DNA quantified to confirm that the amount of islets per tube was similar between conditions. Glucagon was measured by ELISA (R&D Systems).

### Immunofluorescence

Chromaffin cells were fixed by adding an equal volume of 4% formaldehyde in CMF-PBS to the culture medium and incubating for 20 min at room temperature. Cells were blocked and permeabilized in CMF-PBS containing 2% BSA, 1% fish skin gelatin and 0.02% saponin. Primary antibodies were diluted in blocking solution at 1∶500 (SgII) and 1∶100 (δ SA4). The following secondary antibodies conjugated to Alexa Fluor dyes (Invitrogen) were used at 1∶1000 in blocking solution: goat anti-rabbit IgG 488 and goat anti-mouse IgG 594. Images were acquired using a Zeiss LSM 510 confocal microscope and 100× oil objective.

### Adrenal Gland Granin Content

*pearl* and *mocha* mice were obtained from the Jackson Laboratory, and *mocha* animals were backcrossed to C57BL/6 to remove *grizzled* and *Pde6b^rd1^* alleles. *Ap3b2* KO mice were obtained from V. Faundez (Emory) and S. Voglmaier (UCSF). Double mutant *pe/pe*; *Ap3b2*−/− mice were generated by crossing *pe/pe*; *Ap3b2*+/− males to *pe*/+; *Ap3b2*+/− females. Adrenal glands from 3–6 week-old mice were homogenized in 150 mM NaCl, 50 mM Tris-HCl, pH 8.0, 1% NP-40, 0.5% sodium deoxycholate, and Complete protease inhibitors (Roche) with 1 mM EGTA and 1 mM PMSF. After sedimentation at 14,000 g to remove nuclei and cell debris, 20–40 µg protein was separated by electrophoresis through polyacrylamide, transferred to nitrocellulose, and the membranes immunoblotted for AP-3δ and SgII, with actin as loading control and the appropriate secondary antibodies conjugated to IRDye800 (Rockland Immunochemicals). The immunoreactivity was quantified by imaging with an Odyssey system (LI-COR Biosciences) and ImageJ (National Institutes of Health), and the signals normalized to actin. For western analysis of the SgII secreted from chromaffin cells, Tyrode's solution was collected, sedimented at 300 *g* for 3 min at 4°C, and the supernatant mixed with SDS-PAGE sample buffer before electrophoresis through polyacrylamide. Chromaffin cells were directly lysed by the addition of sample buffer. In this case, SgII and actin were detected using ECL plus (GE Healthcare).

### qPCR

Total RNA was isolated from PC12 cells and mouse adrenal glands using TRIzol reagent (Invitrogen) according to the manufacturer's instructions. To improve the yield of adrenal RNA, ultrapure glycogen (Invitrogen) was added to the TRIzol as carrier. In addition, adrenal RNA was treated with RNAse-free DNAse I (NEB) to remove contaminating genomic DNA. cDNA was synthesized using oligo(dT) or gene-specific primers and a Transcriptor First Strand cDNA Synthesis kit (Roche). qPCR was performed with SYBR Green (Applied Biosystems) on a Stratagene Mx4000 machine. The following primers were used: rat SgII fwd: 5′-ACAATATAAGACAGAGGAAAATTTT-3′, rev: 5′-TGGATAAGAAGCAGAACTG-3′; rat β-actin fwd: 5′-CCGTGAAAAGATGACCCAGATC-3′, rev: 5′-CAGGGACAACACAGCCTG-3′; mouse CgA fwd: 5′-CCAACCGCAGAGCAGAG-3′, rev: 5′-AGCTGGTGGGCCACCTT-3′; mouse SgII fwd: 5′-AAGTGCTGGAGTACCTCAACC-3′, rev: 5′-TTACATGTTTTCCATGGCCCG-3′; mouse GAPDH fwd: 5′-ATGGTGAAGGTCGGTGTGAAC-3′, rev: 5′-TCCACTTTGCCACTGCAAATG-3′.

### Lysosomal Inhibition

Two days after the second siRNA transfection, PC12 cells were incubated for ∼24 h in complete medium supplemented with vehicle or a cocktail of lysosomal protease inhibitors (Sigma) including (in µM) 10 antipain, 10 leupeptin and 5 pepstatin A. Cells were washed on ice with cold PBS and lysed by the addition of 50 mM Tris-HCl, pH 8.0, 150 mM NaCl, 1% Triton X-100, and Complete protease inhibitors (Roche) plus 10 mM EDTA and 1 mM PMSF. Samples were analyzed by quantitative fluorescent immunoblotting.

### Secretion Assays

Cells were transfected with siRNA (100 nM) on days 1 and 3 after plating, washed 2 days later and incubated in Tyrode's solution containing 2.5 mM K^+^ (basal) or 50 mM K^+^ (stimulated) for 30 min at 37°C. The supernatants were collected, cell lysates prepared as described above, and the samples analyzed by quantitative fluorescent immunoblotting.

### Statistical Analysis

Statistical analysis was performed using the Student's two-tailed t-test unless otherwise indicated.

## Supporting Information

Figure S1 *mocha* chromaffin cells display marked reductions in secreted and cellular SgII. Chromaffin cells were stimulated with 20 µM DMPP in Tyrode's solution, or left unstimulated in Tyrode's alone. After a 15 min incubation at 37°C, supernatants were collected and mixed with SDS-PAGE sample buffer, and cells lysed by the addition of sample buffer. Secreted and cellular SgII were detected by immunoblotting. DMPP clearly induces secretion of SgII from control cells, but not from *mocha* cells. Analysis of cellular SgII shows a marked reduction in *mocha* cells. Cellular actin was used as a loading control.
(TIF)

Figure S2 Glucose tolerance, serum glucagon and islet morphology in *mocha* mice. (A) Control and *mocha* mice were fasted overnight and challenged with glucose (2 mg/g body weight) delivered i.p. *mocha* mice show slightly improved glucose tolerance relative to controls. p<0.02 for the area under the glucose-time curve; n = 8 control and n = 5 *mocha* mice. (B) Control and *mocha* mice display comparable serum glucagon levels after an overnight fast. n = 15 control and n = 10 *mocha* mice. (C) *mocha* mice exhibit normal pancreatic islet morphology as determined by double staining for insulin/glucagon and glucagon/somatostatin. Scale bar indicates 30 µm.
(TIF)
