# Supplementary figures and images for: Widespread Dysregulation of Peptide Hormone Release in Mice Lacking Adaptor Protein AP-3
Source: PLoS Genet. 2013 Sep 26;9(9):e1003812. doi: 10.1371/journal.pgen.1003812 (PMC3784564; doi:10.1371/journal.pgen.1003812)

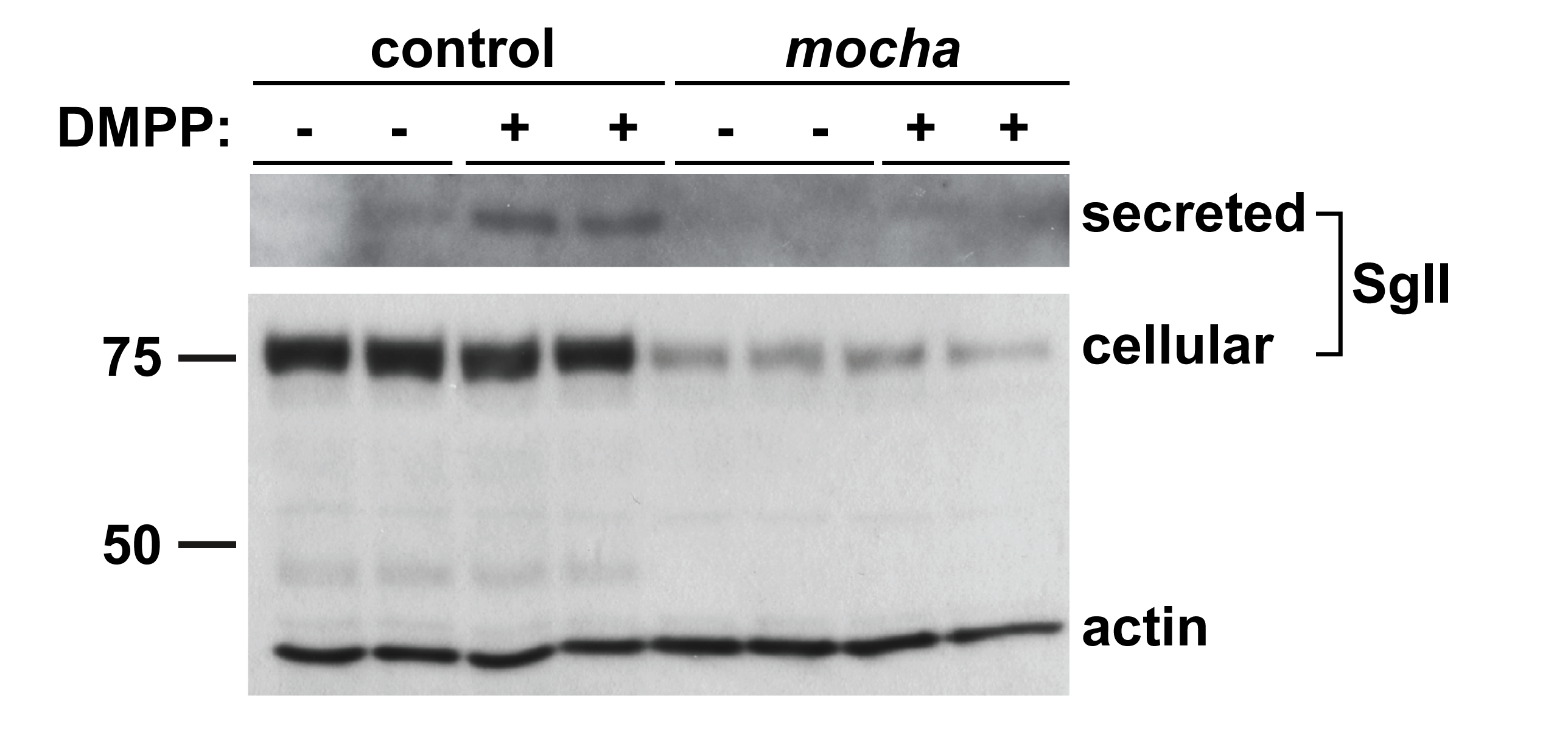

Supplement: Figure S1 — mocha chromaffin cells display marked reductions in secreted and cellular SgII. Chromaffin cells were stimulated with 20 µM DMPP in Tyrode's solution, or left unstimulated in Tyrode's alone. After a 15 min incubation at 37°C, supernatants were collected and mixed with SDS-PAGE sample buffer, and cells lysed by the addition of sample buffer. Secreted and cellular SgII were detected by immunoblotting. DMPP clearly induces secretion of SgII from control cells, but not from mocha cells. Analysis of cellular SgII shows a marked reduction in mocha cells. Cellular actin was used as a loading control. (TIF) [file pgen.1003812.s001.tif]

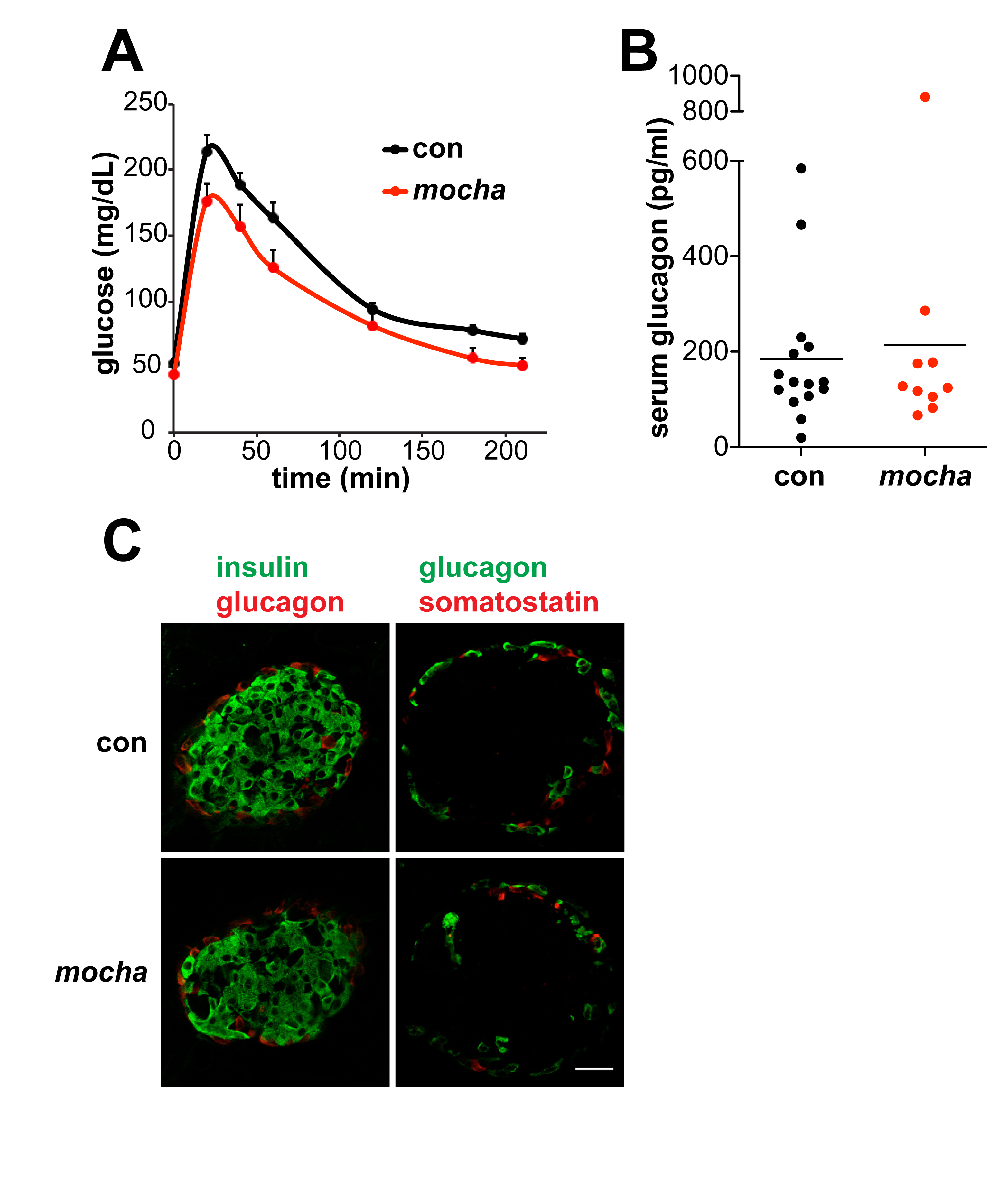

Supplement: Figure S2 — Glucose tolerance, serum glucagon and islet morphology in mocha mice. (A) Control and mocha mice were fasted overnight and challenged with glucose (2 mg/g body weight) delivered i.p. mocha mice show slightly improved glucose tolerance relative to controls. p<0.02 for the area under the glucose-time curve; n = 8 control and n = 5 mocha mice. (B) Control and mocha mice display comparable serum glucagon levels after an overnight fast. n = 15 control and n = 10 mocha mice. (C) mocha mice exhibit normal pancreatic islet morphology as determined by double staining for insulin/glucagon and glucagon/somatostatin. Scale bar indicates 30 µm. (TIF) [file pgen.1003812.s002.tif]
